# Lumbopelvic rhythm during trunk motion in the sagittal plane: A review of the kinematic measurement methods and characterization approaches

**DOI:** 10.7243/2055-2386-3-5

**Published:** 2016

**Authors:** Milad Vazirian, Linda Van Dillen, Babak Bazrgari

**Affiliations:** 1 Department of Biomedical Engineering, University of Kentucky, Lexington, KY, USA; 2 Program in Physical Therapy, Washington University in St. Louis School of Medicine, St. Louis, MO, USA

**Keywords:** Low back pain, disability evaluation, torso, pelvis

## Abstract

Lumbopelvic rhythm during trunk forward bending and backward return has been widely investigated to have a better understanding of the pattern of trunk motion, as used in research on low back disorders. Considerable differences in the methods used to measure, and approaches used to characterize the lumbopelvic rhythm hinder the integration of findings of those studies for further research in the future. Thus, the purpose of this review was to summarize the methods for kinematic measurement as well as their characterization approaches for the lumbopelvic rhythm. PUBMED and CINAHL databases were searched for relevant studies. Several types of instruments were found to be used in the reviewed studies, mostly using markers or sensors, which were placed on different parts of spine, with different definitions to measure the lumbar and pelvic motion. Also, various characterization approaches were found to be used, of which some related to the magnitude, while the others to the timing aspects of lumbopelvic rhythm. Such a characterization was either qualitative or quantitative. In addition, the specified characterization approaches were applied on a sample of trunk kinematics data from our lab to demonstrate differences in the outcomes of these approaches.

## Introduction

Trunk motion in the sagittal plane results from the motions of the lumbar spine and pelvis. The magnitude and timing of such lumbar and pelvic contributions to trunk motion have been investigated extensively for different purposes in the rehabilitation and ergonomic literature under the label of lumbopelvic rhythm (LPR). In general, the timing aspect of LPR has been investigated to obtain insights into the neuromuscular control of trunk motion, and the magnitude aspect of LPR has been investigated to understand the load partitioning within the lower back tissues. Measurement methods and approaches used to characterize timing and magnitude aspects of LPR vary across studies. Efficient integration of earlier research findings related to LPR and choosing the most appropriate characterization approaches for LPR has become a challenging task. To overcome such a challenge, we have summarized the methods used to characterize LPR. This includes a summary of methods used for the collection of kinematic data, as well as a summary of the approaches used to characterize the timing and magnitude aspects of LPR. Finally, we apply various LPR characterization approaches from all categories used in prior research based on our summary, to the kinematic data collected from a research participant in a single trial of trunk motion. The purpose of the application is to demonstrate similarities and differences when LPR is characterized using the different approaches.

## Methods

### Literature review

PUBMED and CINAHL databases were searched for studies including the following keywords in the title or abstract: “lumbopelvic rhythm”, “lumbo-pelvic rhythm” “lumbar-pelvic rhythm”, “spino-pelvic rhythm”, “lumbopelvic coordination”, “lumbo-pelvic coordination”, “lumbar-pelvic coordination”, and “spino-pelvic coordination”. A total of 42 studies were identified. The studies were further screened for inclusion of in-vivo measurements in human participants, and reporting LPR during trunk motion in the sagittal plane. In addition, references of each identified study were also investigated to identify any study that was missed in the database search, adding 12 more studies to the collection. Twenty seven studies (Table 1) met all our criteria, and thus were included in the review. Methods and approaches used to characterize LPR, specifically kinematic measurement methods, as well as approaches used to characterize both the timing and the magnitude aspects of LPR were summarized.

### Kinematic data used for comparison of approaches

Following the literature review, a set of kinematic data was selected from an existing database in our lab that had been obtained from sixty asymptomatic individuals between 20 and 70 years old. The kinematic data included thoracic and pelvic motions in the sagittal plane and were collected during a trunk forward bending and backward return. Participants were instructed to bend forward from an upright position “as fast as possible”. The goal was to reach their maximum comfortable bent posture without any abdominal muscle effort at the end, and then return to the initial upright position. They were instructed to repeat the above motion three times while the thoracic (at T10) and pelvic motions were measured using two magnetic inertial motion trackers (Xsens Technologies, Enschede, Netherlands). Motions of the thorax and pelvis in the sagittal plane were calculated using assumed standing as the reference posture. The lumbar motion was calculated as the difference between the thoracic and pelvic motions. We used the set of kinematic data from the participant with the maximum thoracic motion that was the median of the entire sample for comparison. We examined the timing and the magnitude aspects of LPR based on the approaches in the current review.

## Results

### Kinematic measurements for lumbopelvic rhythm

**Figure 1:**
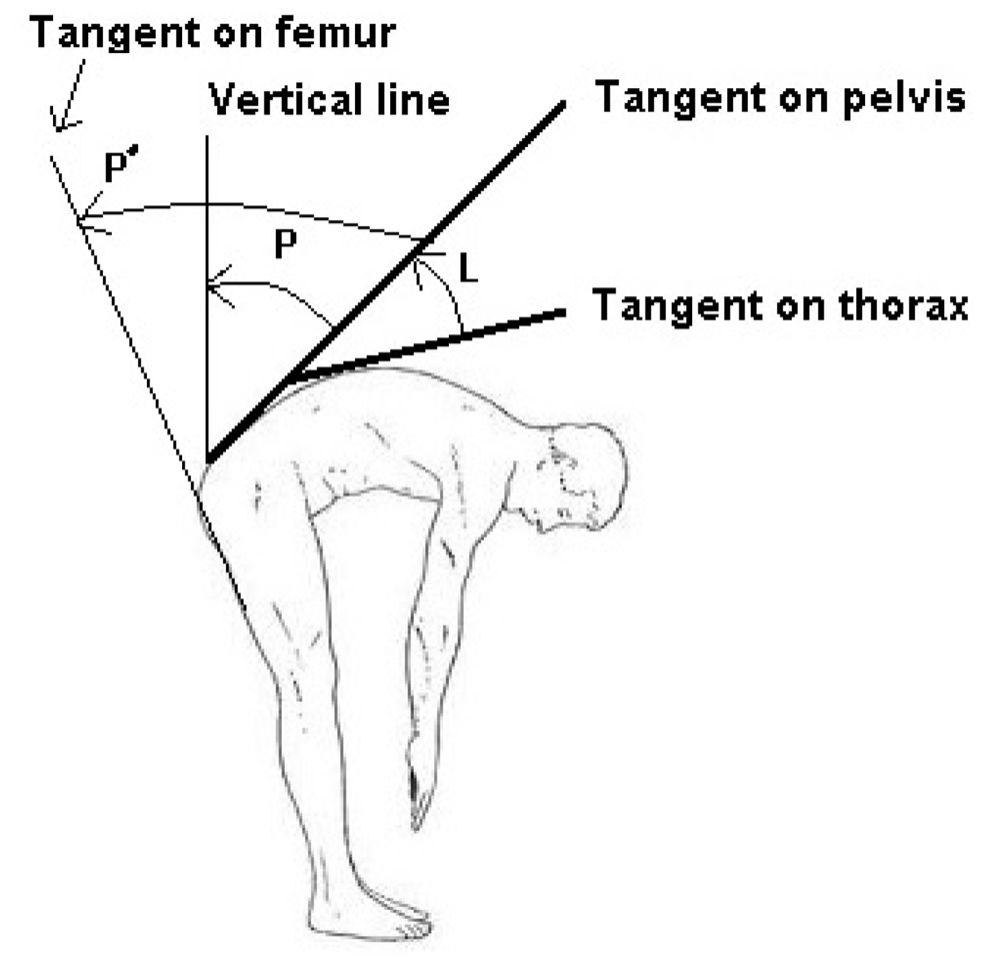
The angles used for calculation of lumbar (L) and pelvic (P: global characterization, P′: local characterization) motion. The changes in angles L and P with time are defined as the lumbar and pelvic motions respectively.

In the studies reviewed, pelvic motion has been characterized as the relative motion of the pelvis with respect to either a local (i.e., thigh) or global (i.e., gravity direction) axis. While the global characterization of pelvic motion represents the contributions of all lower extremity joints to the trunk motion, the local version only represents the contribution of hip joint motion. Lumbar motion generally has been characterized as the relative motion of the thorax with respect to the pelvis in most of the reviewed studies (Figure 1).

Depending on the instrument used for the measurements, joint motions were determined either directly using goniometers, or indirectly by measuring the motion of the segments that constitute the joints using reflective markers or motion sensors. Measurement of a segment motion using inertial or magnetic motion sensors requires attachment of the sensor to one anatomical landmark on the segment. Motion of two or more anatomical landmarks should be tracked (i.e., making a line or a plane) when using markers. Anatomical landmarks that have been used to measure pelvic motion included L5 [1–4], S1 [4–11], S2 [12–14] as well as a plane or line passing through multiple anatomical landmarks on the pelvis and sacrum, for example, a plane defined by markers on the anterior and posterior superior iliac spine [2,15–20]. For thoracic motion (i.e., upper segment of lumbar joint) L1 [1,3,4,9,12–14,19,21,22], T12 [1,2,4–8,12,16,23,24], T11 [17,20], T10 [11], T8 [10], a vector created by markers between T12-L1 [12,14], or a vector created by markers between T11-L1 [17,20] were the anatomical landmarks used. The specific instrumentation and anatomical landmarks used in each study is listed in Table 1. Other than goniometers for direct measurement of lumbar motion, Hasebe et al., [1] used a hand-held, computer-assisted electromechanical mouse device which is able to manually measure the spinal curvature by moving the mouse along the midline of the spine [25]. Pries et al., [4] also used the Epionics SPINE system which consists of two flexible sensor strips with strain gauge sensors along with two accelerometers to measure lumbar spinal shape and motion. The system also measures sacral orientation as a representation of pelvic orientation and motion in the sagittal plane.

### Characterization approaches for lumbopelvic rhythm

Lumbopelvic rhythm refers to the relative pattern of the lumbar and pelvic contributions to trunk motion in the sagittal plane. The aspects of motion of interest include timing, as well as magnitude-related characteristics. The characterization approaches used are mathematical procedures that qualitatively or quantitatively characterize both the timing and magnitude contributions. Similar to differences in the kinematic measurement methods, there have been differences in the approaches used to characterize the timing and magnitude-related aspects of LPR (Table 1).

#### Qualitative approaches for the timing of contribution

**Figure 2:**
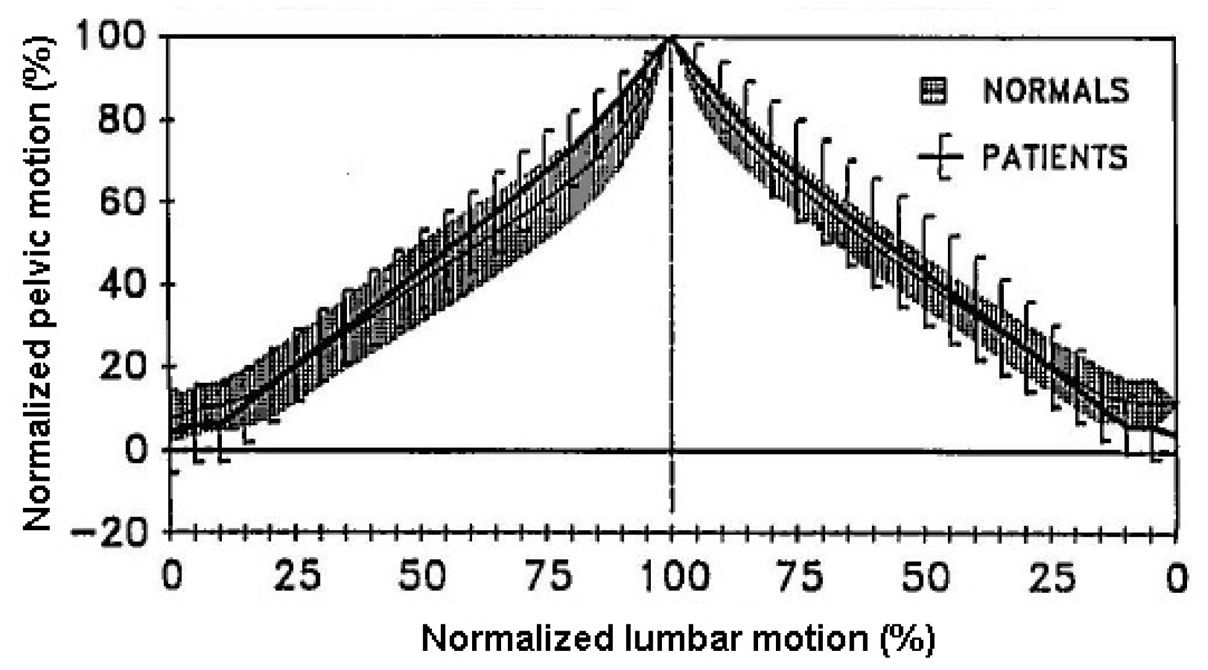
Qualitative characterization for the timing of contribution on the basis of comparison between slopes of curves representing pelvic and lumbar motion. Adopted from [10].

Qualitatively, timing of contribution has been characterized by plots of normalized lumbar or pelvic motion with respect to the other or their sum. Presence of near horizontal or near vertical segments in such a plot would represent respectively minimal or maximal contribution of either the lumbar spine or pelvis to the trunk motion during specific periods. For example, the steep slope of the curve representing the pelvic motion as compared to an almost horizontal curve representing lumbar extension at the start of “Up lift” reported by Nelson et al., [15], suggests a trunk motion primarily started by pelvic motion (Figure 2).

#### Quantitative approaches for the timing of contribution

**Figure 3:**
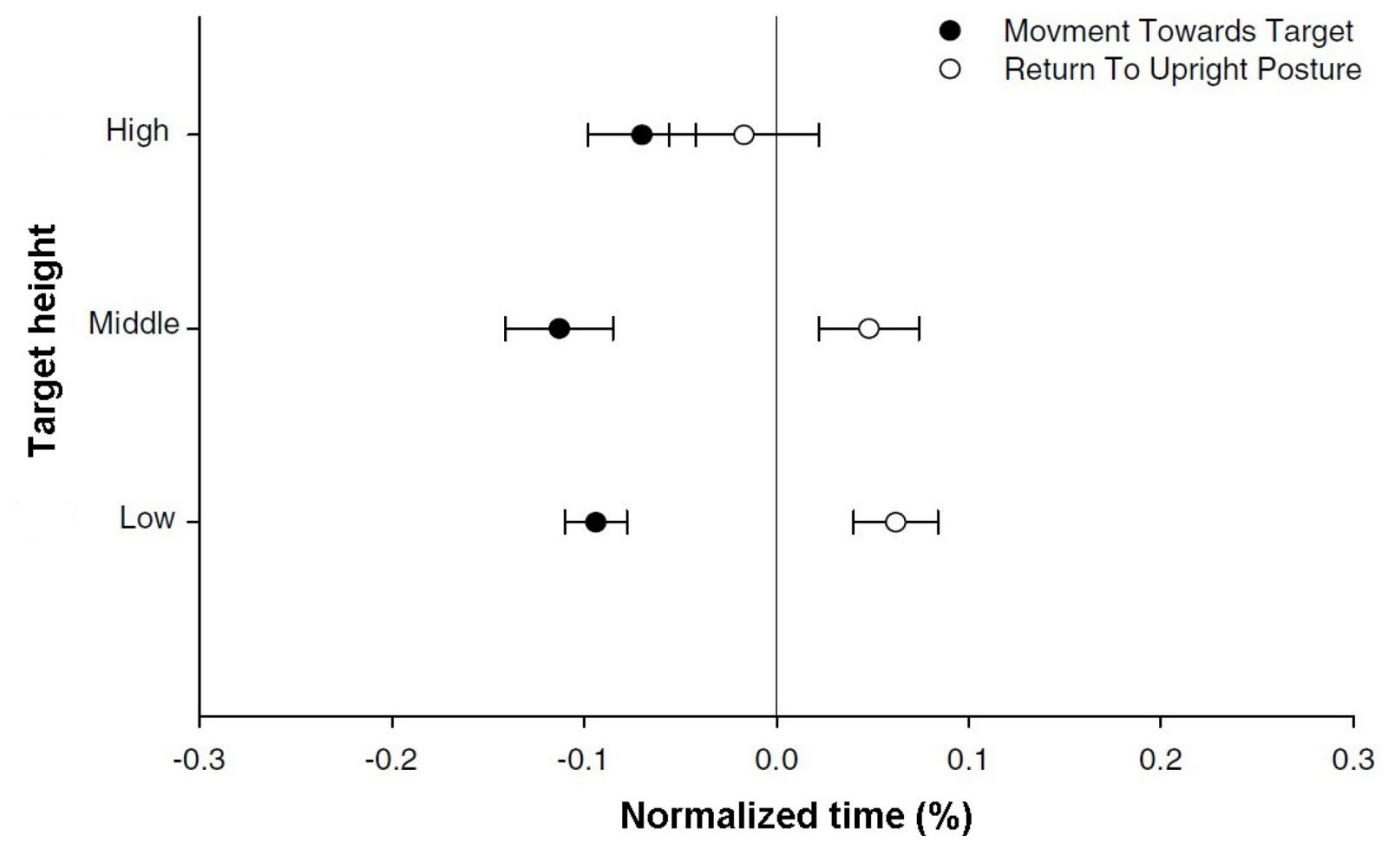
Quantitative results for the differences in timing of contribution between lumbar and pelvic motion when lifting an object from different heights. The time difference is normalized to total movement time and negative values indicate the lumbar spine motion is ahead of pelvis motion.

Three different approaches were identified for quantitative characterization of the timing of contribution. These approaches include the following methods: (1) critical points, (2) cross-correlation, and 3) relative phase. In the critical points method, a time difference is calculated between different event times (e.g., onset, termination, maximum displacement, or maximum velocity) of lumbar and pelvic motion [17,26]. Using this approach, Thomas et al., [26] compared the onset delays of the lumbar spine with respect to the pelvis in trunk forward bending and backward return. The onset delays were examined between reaching tasks to targets at low, middle, and high height levels, and were reported as percentages of the total motion time, as depicted in Figure 3.

For the cross-correlation method, the lumbar and pelvic motion are cross-correlated by determining a time lag (phase) that is associated with the maximum correlation between the temporal variations of both lumbar and pelvic motion during the task [21,22]. The time lag is an indication of the amount of time that one signal, in this case the kinematics of the pelvis or lumbar spine, is ahead or behind the other signal. For example, Lee et al., [21] observed that lumbar motion relative to the pelvic motion had a mean (SD) time lag of −0.01 (0.04) and 0.02 (0.06) seconds when pelvic motion was calculated locally relative to left and right thigh, respectively. The negative sign of time lag indicated that the lumbar spine was behind the pelvis and vice versa.

Finally, in the relative phase method, a phase plane is initially generated for the lumbar and pelvic motion using normalized velocity and displacement. The normalization procedure for the velocity is implemented by dividing the velocity of each instant to the maximum absolute velocity in the range. The displacement is normalized by setting the minimum and maximum values respectively to −1 and 1. The phase planes are in a closed loop form, and the phase angle for each data point is calculated as the angle of the line connecting the point to the center of the plot with respect to the horizontal (i.e., displacement) axis [27]. The difference between the phase angles of lumbar and pelvic motion at each time instant is obtained from their phase planes, which results in a continuous relative phase curve. The relative phase is then calculated as the average of such continuous relative phase curve over the total trunk motion or any portion of the total trunk motion [27]. A relative phase of 0 represents a perfectly synchronous (in-phase) contribution from the lumbar spine and pelvis. A relative phase of π radians (180 degrees) represents a perfectly asynchronous (out-of-phase) contribution from the lumbar spine and pelvis. For example, Hu et al., [8] observed that the mean relative phase for return from the fully bent posture to the standing posture without and with a 20 pound load in the hands is 0.45 and 0.23 radians, respectively. These findings indicate that the lumbar and pelvic motions are more in-phase with versus without the load.

#### Qualitative approaches for the magnitude of contribution

**Figure 4:**
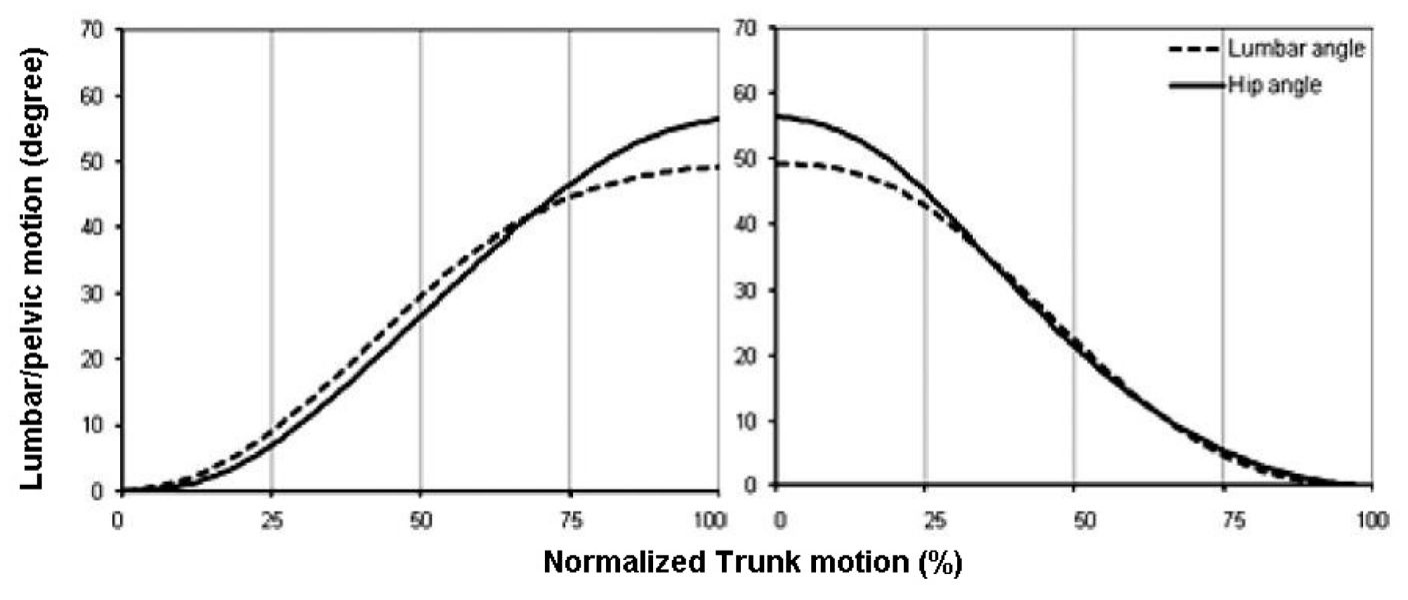
Plotting lumbar and pelvic motions as functions of normalized trunk motion allows a qualitative comparison of the contribution of lumbar and pelvis motion to trunk motion. For any given instant of motion, when the lumbar curve is above (below) the pelvic curve, it means that up to that point in time the total contribution of lumbar to trunk motion has been larger (smaller) than pelvis. The pelvic contribution in example shown here [23] is characterized locally with respect to thigh (i.e., hip flexion).

Qualitatively, magnitudes of contribution were characterized by investigation of curves representing percent of trunk motion in the sagittal plane provided by either lumbar or pelvic motion. Curves representing the absolute lumbar or pelvic motion compared to absolute or normalized trunk motion also were used. For any given instant of motion, if the lumbar curve is above (below) the pelvic curve it means that up to that point in time the total contribution of lumbar to trunk motion has been larger (smaller) than the pelvis. As an example, Kim et al., [23] studied LPR in a healthy group of participants, and observed that the curve of pelvic motion is higher than the curve of lumbar motion in the late and early stages of the trunk forward bending and backward return, respectively. The pattern of the magnitude of contribution was the same in other parts of the trunk motion. So, the authors suggested that the total contribution of pelvis was larger than the total contribution of lumbar spine in the late and early stages of trunk forward bending and backward return, but their total contributions were almost equivalent elsewhere (Figure 4).

#### Quantitative approaches for the magnitude of contribution

**Figure 5:**
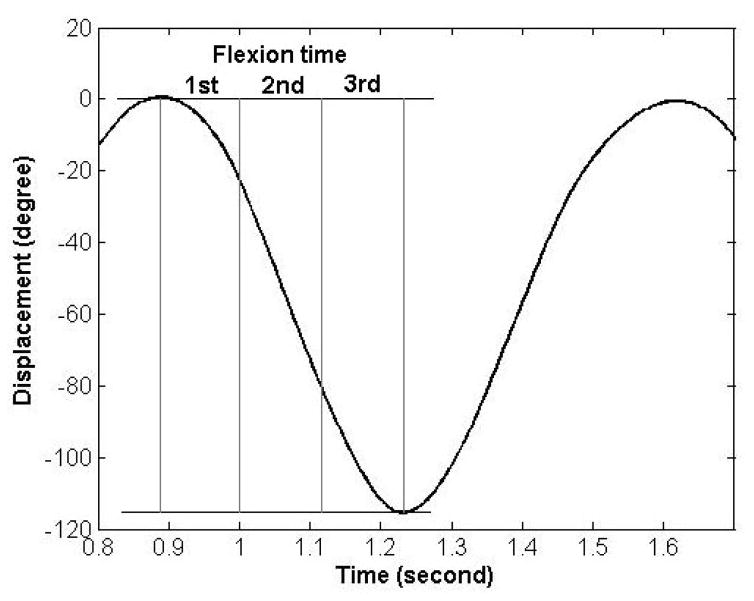
Phillips et al reported the ratios of mean lumbar to mean pelvic motion, as lumbopelvic ratios, for three equal sized time-windows during the forward bending phase of the motion. The figure has been reproduced using data obtained from authors [28].

The magnitude of lumbar spine contribution has been characterized quantitatively by calculating ratios of average lumbar motion to average pelvic motion (i.e., lumbopelvic ratio) during several different time intervals over the period of a specific trunk motion. The time intervals were either a given percent of total motion time (e.g., 25% of bending time) or the time required to complete a given percent of actual trunk motion (e.g., 25% of trunk motion) (Figure 5). Compared to the qualitative approaches that offer information related to the total contribution, lumbopelvic ratios indicate to the relative contribution of lumbar and pelvic motion to trunk motion over the studied time window. For example, Phillips et al., [28] observed that the mean (SD) of the lumbopelvic ratio for a group of participants was 4.04 (5.20), 0.54 (0.08) and 0.47 (0.15) for the first, second and the third time intervals of trunk forward bending at a self-selected pace.

The ratio of the lumbar to pelvic range of motion (i.e., lumbopelvic ratio over the entire trunk range of flexion) also has been used to characterize the magnitude of contribution. It should be kept in mind, however, that such a ratio represents the relative lumbar and pelvic contribution to trunk motion only at the end range of trunk motion, and does not offer any information related to relative contribution at other time points during the motion.

### Characterization of lumbopelvic rhythm: A sample experiment

To provide a comparison of results related to timing and magnitude of contribution obtained from the approaches reviewed in the previous section, we applied the approaches to a set of kinematic data that were obtained from one participant in our laboratory.

#### Qualitative approaches for the timing of contribution

**Figure 6:**
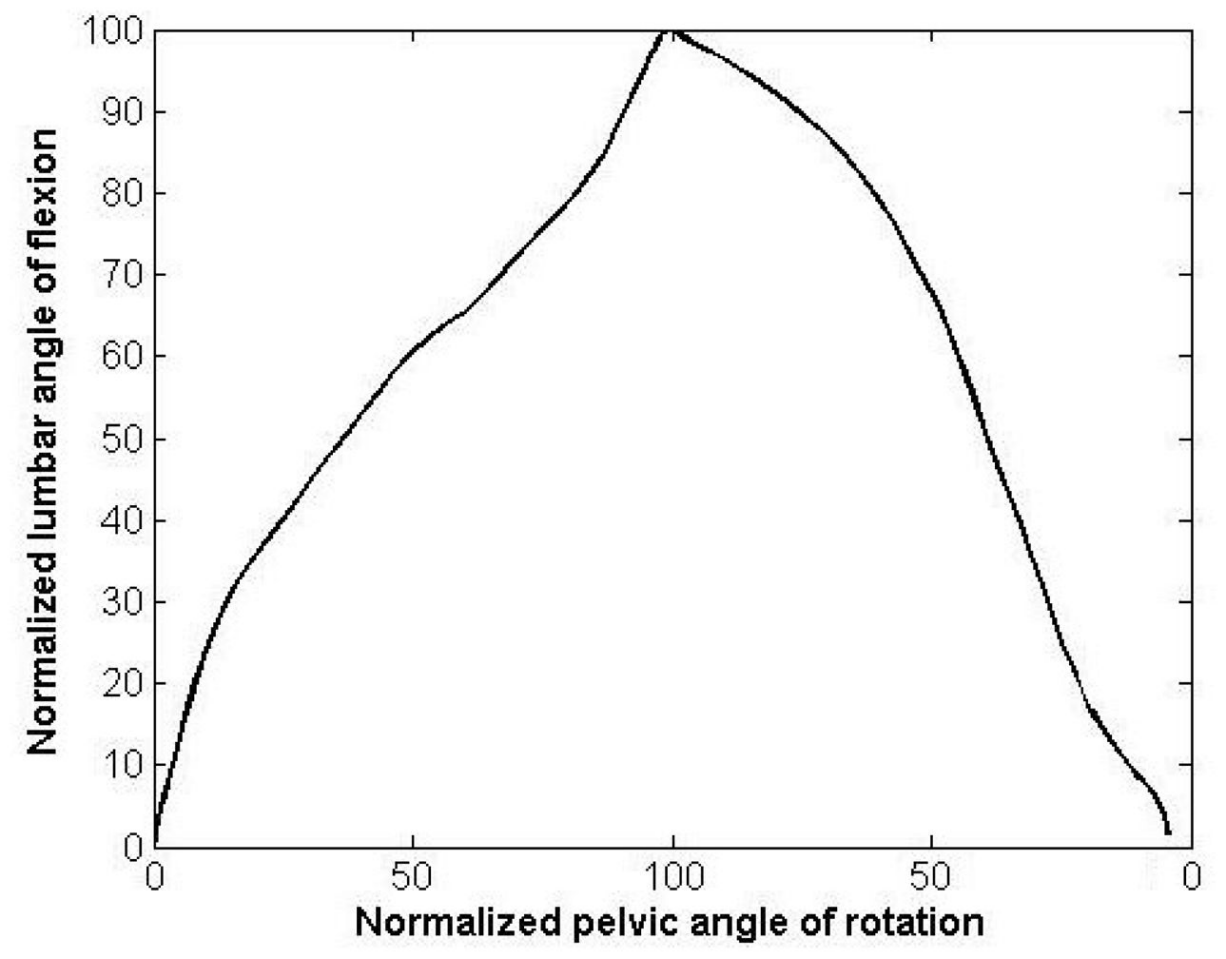
Qualitative characterization of the timing of contribution can be done on the basis of comparison between slopes of curves representing pelvic and lumbar motion. The absence of near vertical or horizontal regions in the curve suggests that pelvic and lumbar motion simultaneously contributed to the trunk motion.

The lumbar and pelvic motion were normalized to their maximum value in the trunk forward bending and backward return cycle. The normalized values for the lumbar spine and pelvis then, were plotted against each other (Figure 6). It can be seen that there is no pure horizontal or vertical part in the curve, suggesting that the lumbar spine and pelvis are contributing to the motion simultaneously across the movements.

#### Quantitative approaches for the timing of contribution

**Figure 7:**
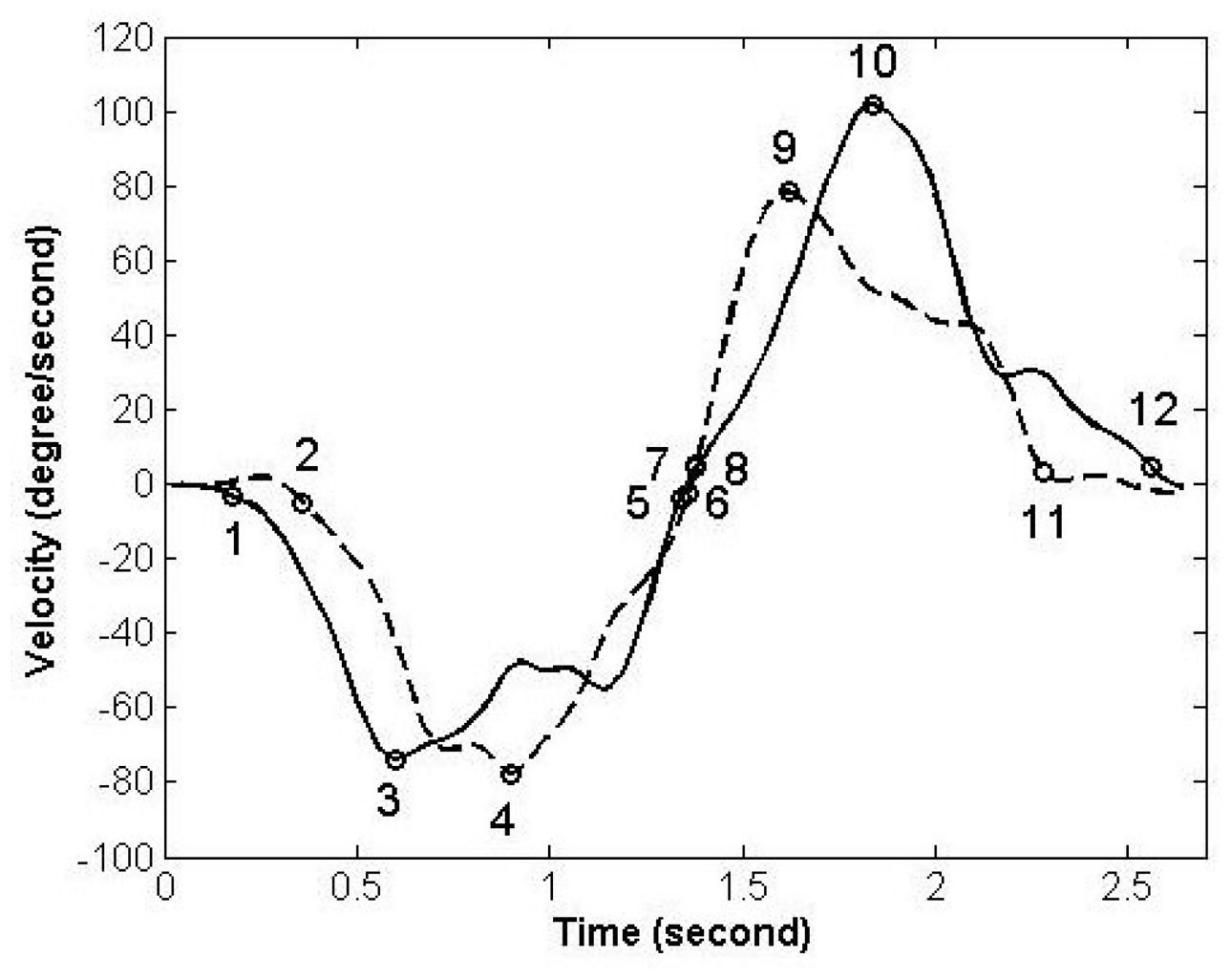
Quantitative characterization of timing of contribution using the Critical Point Method [17]. The timing of contribution is characterized by comparing motion onsets: point 1 (8) for lumbar and point 2 (7) for pelvic motion during forward bending (backward return); motion termination: point 5 (12) for lumbar and point 6 (11) for pelvic motion during forward bending (backward return); and times of peak velocity: point 3 (10) for lumbar and point 4 (9) for pelvic motion during forward bending (backward return).

Using the critical point method, the time differences in the motion onset, peak velocity, and termination of motion between lumbar and pelvic motion were estimated (Figure 7). The time differences were respectively 0.18, 0.30, 0.02 sec in the trunk forward bending, and 0, −0.22 and −0.28 in the backward return when assessed using the time event of the motion onset, peak velocity, and termination of motion. The negative sign indicates that the pelvic motion was ahead of the lumbar motion and vice versa. The time of motion onset and termination for the lumbar spine and pelvis in each phase of motion was specified as the time when the velocity of the lumbar spine or pelvis reaches 0.05 of the peak velocity. The cross-correlation method was performed using a customized program written in MATLAB (Mathworks, Natick, MA. USA) software. We found the time lag to be 0.10 and −0.14 seconds for trunk forward bending and backward return, respectively. The negative sign indicates the pelvis was ahead of the lumbar spine and vice versa.

**Figure 8:**
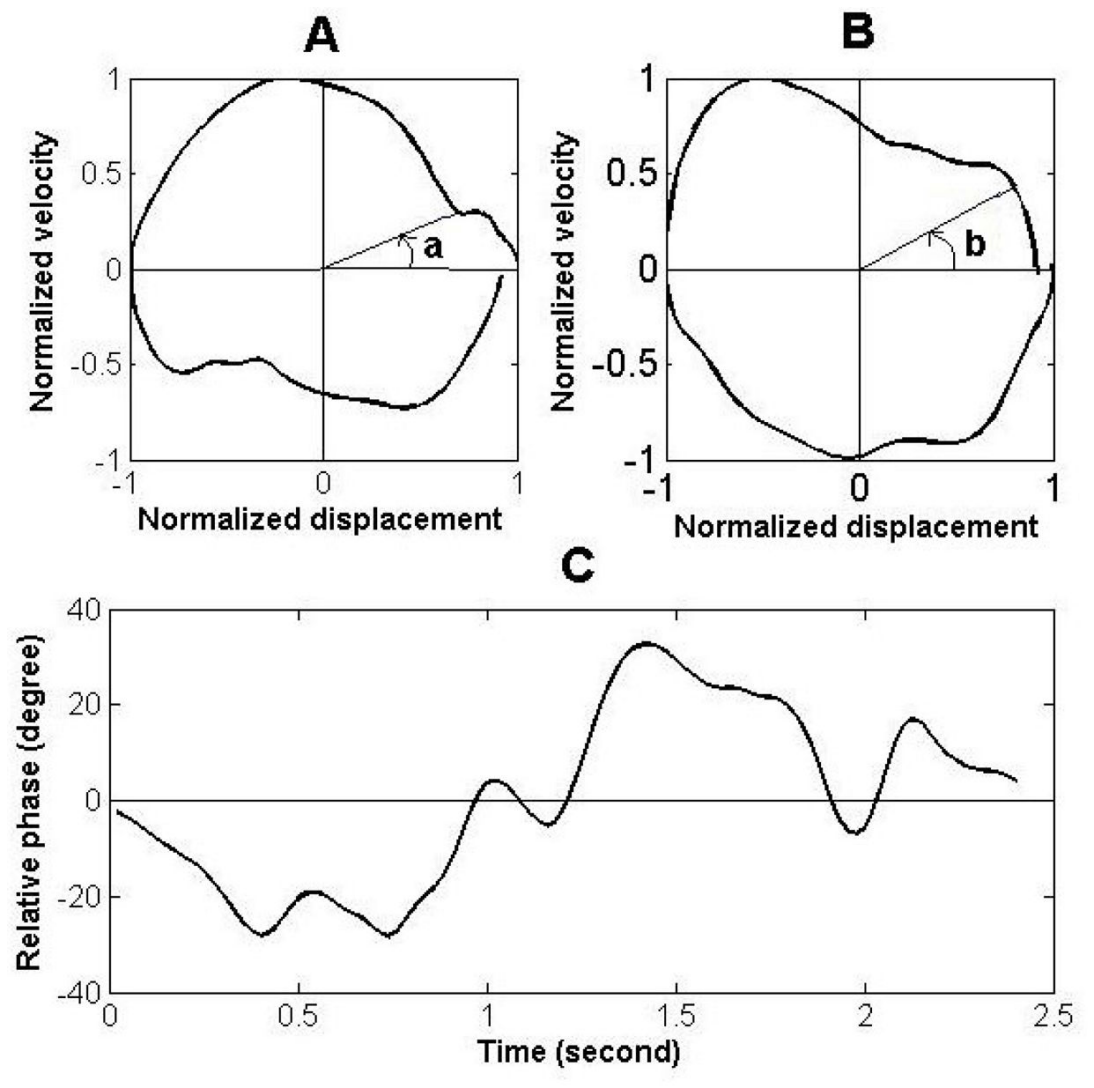
The phase planes for the lumbar spine (**A**) and pelvis (**B**), and the curve of continuous relative phase (**C**) for a sample trial of forward bending and backward return. The angles “a” and “b” represent the phase angle of the lumbar spine and pelvis, respectively.

Finally, to study the timing aspect of LPR using the relative phase method, the phase planes of lumbar and pelvic motions were initially developed as explained above. The continuous relative phase for each time instant subsequently was calculated by subtracting the pelvic phase angle from the lumbar phase angle at that time instant (Figure 8).

The average relative phase for the trunk forward bending and backward return were 0.18 and −0.24 radians respectively. The negative relative phase indicates that the phase of pelvic motion was ahead of the phase of lumbar motion.

#### Qualitative approaches for the magnitude of contribution

**Figure 9:**
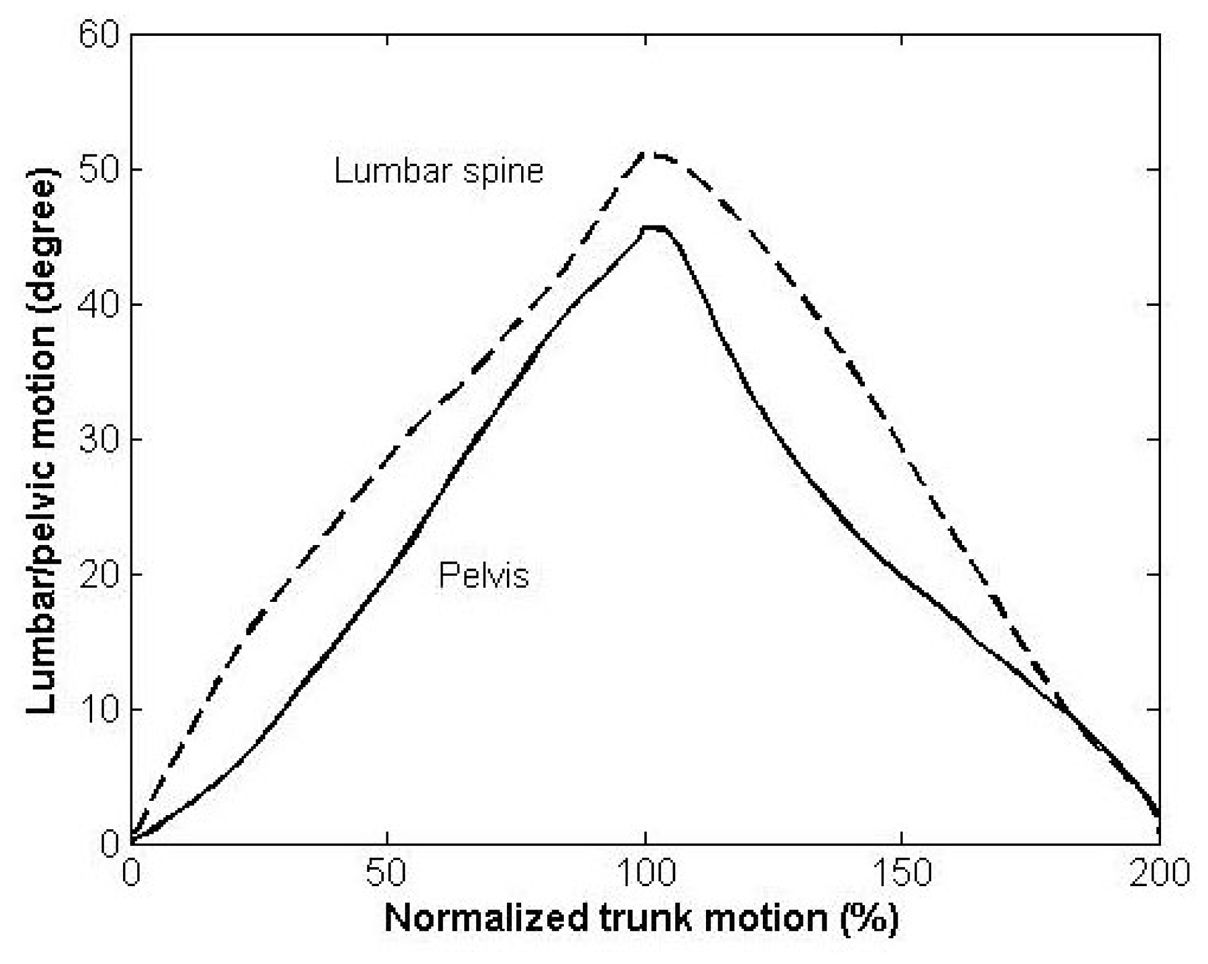
On the basis of a qualitative comparison, our results indicate that total lumbar contribution was larger than total pelvic contribution throughout the motion.

The lumbar and pelvic motions at each percent of the total trunk motion in the trunk forward bending (0 to 100%) and backward return (100% to 200%) were plotted (Figure 9). Attention to this figure reveals that for most of the motion, except toward the end of backward return, the total contribution of lumbar to trunk motion was larger than pelvic contribution.

#### Quantitative approaches for the magnitude of contribution

The lumbopelvic ratio for four equal time intervals were 1.95, 0.95, 0.68 and 1.09 during the trunk forward bending, and 0.49, 1.12, 1.95 and 1.32 during the backward return. A lumbopelvic ratio of larger (smaller) than one for a given time window indicates that the amount of lumbar contribution was larger (smaller) than pelvic contribution during that time window.

## Conclusion

Studies of LPR were reviewed and their methods for kinematic measurement and characterization approaches for LPR were summarized. Measurement of kinematics primarily was performed using markers or sensors. Across studies, there were some differences in anatomical landmarks used to measure lumbar and pelvic motions. The characterization approaches for LPR included both qualitative and quantitative approaches and provided information about the timing or magnitude-related aspects of LPR. All quantitative approaches used to assess the timing aspect of LPR of our sample data indicated that the lumbar spine was ahead (behind) of the pelvis during the forward bending (backward return) phase of the trunk motion. However, the qualitative approach for timing aspect of LPR was not clear on the time difference between the lumbar and pelvic motions, suggesting both contributing simultaneously. The quantitative approach for the magnitude aspect of LPR provided information related to the average amounts of the lumbar and pelvic contributions over certain time windows whereas the qualitative approach provides the total contribution from the starting point.

Although the suitability of each of the summarized approaches needs to be evaluated based on the specific research or clinical question of interest, it is expected that the current review would provide a starting point for such a selection process.

## Figures and Tables

**Table 1:** List of the reviewed studies.

Article	Instruments	Pelvic motion	Lumbar motion	Characterization approaches for LPR
Paquet et al., 1994	Electrogoniometer (JS)	hip flexion (L)	Change of the angle between T8 and S1	Timing: Plot of hip vs. lumbar motion, normalized to their maximum Magnitude: Absolute displacement of the hip and lumbar spine
Gracovetsky et al., 1995	Infrared lightemitting diodes (M)	Rotation of the line normal to the plane made by markers on the iliac crests and sacrum (G)	Rotation of the best fit line through the markers on the thoracolumbar spine	Magnitude: Absolute displacement of the hip and lumbar spine
Nelson et al., 1995	3-Space Tracker System (SS)	Sacral rotation (G)	Rotation of the best fit line passing through the whole thoracolumbar spine	Timing: Plot of the lumbar and pelvis motion vs. gross trunk motion normalized to their maximum
Esola et al., 1996	Opteoelectric motion analysis system (M)	Rotation of S2 relative to the posterior midline of thigh (L)	Rotation of T12-L1 segment relative to S2	Magnitude: Lumbar to hip motion ratio for intervals of 0–30, 30–60 and 60–90 degrees & Lumbar to hip motion ratio for each 25% of total duration
McClure et al., 1997	Opteoelectric motion analysis system (M)	Rotation of S2 relative to the posterior midline of thigh (L)	Rotation of T12-L1 segment relative to S2	Magnitude: Lumbar to hip motion ratio for each 25% of extension
Porter & Wilkinson, 1997	3-Space Tracker System (SS)	Sacral rotation relative to the lateral femoral condyle (L)	Rotation of T12 relative to the sacrum	Magnitude: Contribution of the lumbar spine and hip to the movement at 15°, 30°, 60°, 90°, and 120°
Tully & Stillman, 1997	Videotape (M)	Rotation of the line from mid-PSIS to ASIS relative to the line from 2/3 Th to LFC (L)	Rotation of T10-T12 segment relative to the line from mid-PSIS to ASIS	Magnitude: Displacement curves of the hip and spine
Granata & Sanford, 2000	Electromagnetic sensors (SS)	Rotation of S1 (G)	Rotation of T12 relative to S1	Timing: Lumbar vs. pelvic motion plot Magnitude: Lumbar to pelvic motion ratio for intervals of 0–30, 30–60 and 60–90 degrees
Lariviere et al., 2000	Video cameras (M)	Sacral rotation (G)	Rotation of the thoracic vertebrae relative to the sacrum	Timing: Mean, standard deviation and maximum of the continuous relative phase
Lee & Wong, 2002	3SPACE Fastrak (JS)	Sacral rotation relative to the lateral aspect of the left and right thighs (L)	Rotation of L1 relative to sacrum	Timing: Time lag (maximum cosscorrelation between the lumbar and pelvic velocity curves) Magnitude: Absolute displacement of the hip and lumbar spine
Wong & Lee, 2004	3SPACE Fastrak (JS)	Sacral rotation relative to the posterior aspect of the left and right thighs (L)	Rotation of L1 relative to sacrum	Timing: Time lag (maximum coss-correlation between the lumbar and pelvic motion velocity curves) Magnitude: Absolute displacement of the hip and lumbar spine
Pal et al., 2007	3-D Motion Analysis System (M)	Rotation of the line from the mid of ASISs to the mid of PSISs relative to the line from 1/3 thigh to LFE (L)	Rotation of the line between T11 and L1 relative to Line between the two ASISs and PSISs	Timing: Time of initiation of each and time to reach the peak velocity Magnitude: Absolute displacement of the hip and lumbar spine
Thomas et al., 2007	Magnetic based kinematic system (SS)	Sacral rotation relative to the right thigh (L)	Rotation of T1 relative to sacrum	Timing: Movement latencies for the initiation, peak and termination of motion Magnitude: Lumbar to hip motion ratios for the quartiles of movement
Milosavljevic et al., 2008	3-D Motion Analysis System (M)	Rotation of the line between the two ASISs and PSISs relative To the line from 1/3 thigh to LFE (L)	Rotation of the line between T11 and L1 relative to Line between the two ASISs and PSISs	Timing: Time of initiation of each and time to reach the peak velocity
van Wingerden et al., 2008	Video (M)	Rotation of the line from sacrum to anterior superior iliac spine (G)	Rotation of the line from L1 to 7cm above relative to the line from sacrum to anterior superior iliac spine	Magnitude: Slopes coming from the regression between displacements of the spine and the total trunk displacement in the 1st and 3rd intervals
Silfies et al., 2009	Electromagnetic tracking device (SS)	Rotation of S2 relative to the lateral epicondyle (L)	Rotation of L1 relative to S2	Timing: Mean absolute relative phase (MARP) and deviation phase (DP)
Kim et al. 2013	3-D Motion Capture System (M)	Pelvic rotation relative to the femur (L)	Rotation of T12 relative to the pelvis	Magnitude: Absolute displacement of the hip and lumbar spine & Lumbar to hip motion ratios for the quartiles of movement
Hasebe et al., 2013	Video (M)	Sacral rotation (G)	Rotation of L5 relative to L1	Magnitude: Lumbar to hip motion ratio for three intervals of forward bending
Hu et al., 2014	Magnetic field based motion tracking system (SS)	Rotation of S1 (G)	Rotation of T12 relative to S1	Timing: Continuous relative phase for each 25% of the trunk motion time
Iwasaki et al., 2014	Electrogoniometers (JS)	Sacral rotation	Rotation of L5 relative to L1	Timing: Plot of normalized lumbar and pelvic motion vs. the normalized trunk duration of motion
Lariviere et al., 2014	3D-motion system comprising inertial sensors (SS)	Sacral rotation	Rotation of the thoracic vertebrae relative to the sacrum	Timing: Mean, standard deviation and maximum of the continuous relative phase
Phillips et al., 2014	Motion capture system (M)	Not available	Not available	Magnitude: Lumbar to pelvic motion ratios for the quartiles of movement
Tafazzol et al., 2014	Inertial and magnetic sensors (SS)	Rotation of S1	Rotation of L1 relative to S1	Timing: Normalized pelvic vs normalized lumbar motion Magnitude: Lumbar to pelvic motion ratio for each 10% increment of the motion
Hu & Ning, 2015 (A)	3D, magnetic field based motion tracking system (SS)	Rotation of S1 (G)	Rotation of T12 relative to S1	Timing: Normalized pelvic motion vs normalized lumbar motion Magnitude: Lumbar to pelvic motion ratio for each 10% increment of the motion
Hu & Ning, 2015 (B)	3D, magnetic field based motion tracking system (SS)	Rotation of S1 (G)	Rotation of T12 relative to S1	Timing: Continuous relative phase for each 25% of the trunk motion time
Pries et al., 2015	Epionics SPINE system (JS)	Sacral rotation (G)	Change in the lumbar lordosis	Magnitude: Lumbar to pelvic motion ratio for each point of the motion & Lumbar to pelvic motion ratio for the early, middle and late stages of motion, as well as the total motion
Vazirian et al., *Under Review*	Magnetic-inertial motion trackers (SS)	Pelvic rotation (G)	Rotation of T10 relative to pelvis	Magnitude: Lumbar to thoracic motion ratio for four quarters of the motion

Summary of letter under each column is given in the footnote of the table. Instrument column: JS: joint sensor, SS: segment sensor, M: Marker. Pelvic motion column: L: local, G: global
